# 2-(1-Adamant­yl)-4-bromo­anisole at 123 K

**DOI:** 10.1107/S1600536808016772

**Published:** 2008-06-07

**Authors:** Xiang-Wei Cheng

**Affiliations:** a Zhejiang Police College Experience Center, Zhejiang Police College, Hangzhou 310053, People’s Republic of China

## Abstract

In the title compound [systematic name: 2-(1-adamantyl)-4-bromo-1-­methoxy­benzene], C_17_H_21_BrO, two weak intra­molecular C—H⋯O hydrogen bonds influence the mol­ecular conformation. The crystal packing exhibits C—H⋯π inter­actions, with a relatively short inter­molecular C⋯*Cg* contact of 3.568 (5) Å, where *Cg* is the centroid of the benzene ring. The crystal studied exhibited inversion twinning.

## Related literature

For related crystal structures, see: Nordman & Schmitkons (1965 ▶); Amoureux *et al.* (1980 ▶); Amoureux & Bee (1980 ▶); Pouwer *et al.* (2007 ▶). For general background, see: Chomienne *et al.* (1994 ▶). For synthesis, see: Antibes *et al.* (1988 ▶).
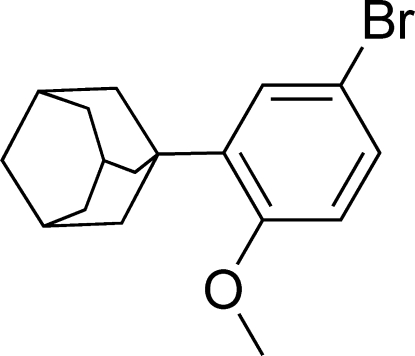


## Experimental

### 

#### Crystal data

C_17_H_21_BrO
*M* *_r_* = 321.25
Orthorhombic, 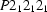

*a* = 7.3815 (11) Å
*b* = 13.2067 (19) Å
*c* = 15.067 (2) Å
*V* = 1468.8 (4) Å^3^
*Z* = 4
Mo *K*α radiation
μ = 2.79 mm^−1^
*T* = 123 (2) K
0.30 × 0.26 × 0.25 mm

#### Data collection

Bruker SMART CCD area-detector diffractometer
Absorption correction: multi-scan (*SADABS*; Bruker, 2002 ▶) *T*_min_ = 0.438, *T*_max_ = 0.492
12697 measured reflections
2580 independent reflections
2279 reflections with *I* > 2σ(*I*)
*R*_int_ = 0.049

#### Refinement

*R*[*F* ^2^ > 2σ(*F* ^2^)] = 0.040
*wR*(*F* ^2^) = 0.106
*S* = 0.86
2580 reflections
172 parameters
H-atom parameters constrained
Δρ_max_ = 0.51 e Å^−3^
Δρ_min_ = −0.59 e Å^−3^
Absolute structure: Flack (1983 ▶), with 1072 Friedel pairs
Flack parameter: 0.340 (15)

### 

Data collection: *SMART* (Bruker, 2002 ▶); cell refinement: *SAINT* (Bruker, 2002 ▶); data reduction: *SAINT*; program(s) used to solve structure: *SHELXS97* (Sheldrick, 2008 ▶); program(s) used to refine structure: *SHELXL97* (Sheldrick, 2008 ▶); molecular graphics: *SHELXTL* (Sheldrick, 2008 ▶); software used to prepare material for publication: *SHELXTL*.

## Supplementary Material

Crystal structure: contains datablocks global, I. DOI: 10.1107/S1600536808016772/cv2416sup1.cif

Structure factors: contains datablocks I. DOI: 10.1107/S1600536808016772/cv2416Isup2.hkl

Additional supplementary materials:  crystallographic information; 3D view; checkCIF report

## Figures and Tables

**Table 1:** Hydrogen-bond geometry (Å, °) *Cg* is the centroid of the benzene ring.

*D*—H⋯*A*	*D*—H	H⋯*A*	*D*⋯*A*	*D*—H⋯*A*
C9—H9*A*⋯O1	0.99	2.35	3.003 (5)	123
C17—H17*B*⋯O1	0.99	2.35	3.004 (4)	123
C4—H4⋯*Cg*^i^	0.95	2.66	3.568 (5)	161

Symmetry code: (i) 
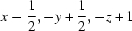
.

**Fig. 1.**
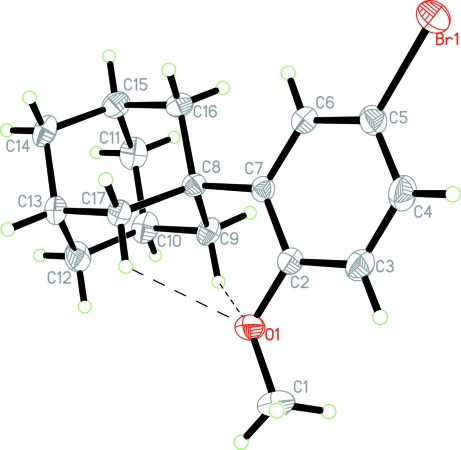
Molecular structure of the title compound, showing 30% probability displacement ellipsoids and the atomic numbering.

## supplementary crystallographic information

### Comment

The molecule of adamantane has high symmetry, Td, and adamantane crystallizes in the highest space group, Fm3 m (Nordman & Schmitkons, 1965; Amoureux *et al.*, 1980; Amoureux & Bee, 1980). In view of the development of crystal structure systems and the design of organic crystals, it is of interest to study the effects of some simple functional substituents having hydrogen-bonding ability on the symmetry of the crystals of adamantane derivatives. The title compound is an important intermediate of adapalene, which is a new synthetic retinoid of the naphthoic acid series, and was developed for the topical treatment of Acne vulgaris and prevention of some forms of cancer, including the acute promyelocytic leukaemia (Chomienne *et al.*, 1994). Here we report the crystal structure of the title compound (Fig. 1).

In the title compound, the structural parameters of the adamantyl are closely comparable to those found in reported molecule (Pouwer *et al.*, 2007). The C atoms of the adamantine moiety have C*sp*^3^ hybridized orbitals, with C—C—C angles in the range 106.6 (4)–111.6 (4)°. The methoxy group and bromo group are coplanar with the benzene ring.

It is of note that the O atoms of the methoxy group participates in formation of two intramolecular C—H···O interactions,and both intramolecular C—H···O interactions are nearly the same (Table.1). Meanwhile, in the crystal structure, an intermolecular C—H···π interaction involving the benzene ring (with the centroid Cg) is observed (Table 1).

### Experimental

The title compound was prepared according to the literature method (Antibes *et al.*, 1988). Crystals suitable for X-ray analysis were obtained by slow evaporation of an 2-propanol solution at 295 K.

### Refinement

H atoms were positioned geometrically (C—H = 0.95–0.99 Å) and refined using a riding model, with *U*_iso_(H) = 1.2–1.5*U*_eq_(C).

### Crystal data

**Table:**

C_17_H_21_BrO	*F*_000_ = 664
*M**_r_* = 321.25	*D*_x_ = 1.453 Mg m^−^^3^
Orthorhombic, *P*2_1_2_1_2_1_	Mo *K*α radiation λ = 0.71073 Å
Hall symbol: P 2ac 2ab	Cell parameters from 2580 reflections
*a* = 7.3815 (11) Å	θ = 2–25.0º
*b* = 13.2067 (19) Å	µ = 2.79 mm^−^^1^
*c* = 15.067 (2) Å	*T* = 123 (2) K
*V* = 1468.8 (4) Å^3^	Block, colourless
*Z* = 4	0.30 × 0.26 × 0.25 mm

### Data collection

**Table:**

Bruker SMART CCD area-detector diffractometer	2580 independent reflections
Radiation source: fine-focus sealed tube	2279 reflections with *I* > 2σ(*I*)
Monochromator: graphite	*R*_int_ = 0.049
*T* = 123(2) K	θ_max_ = 25.0º
φ and ω scans	θ_min_ = 2.1º
Absorption correction: multi-scan(SADABS; Bruker, 2002)	*h* = −8→8
*T*_min_ = 0.438, *T*_max_ = 0.492	*k* = −15→14
12697 measured reflections	*l* = −17→17

### Refinement

**Table:**

Refinement on *F*^2^	Hydrogen site location: inferred from neighbouring sites
Least-squares matrix: full	H-atom parameters constrained
*R*[*F*^2^ > 2σ(*F*^2^)] = 0.040	*w* = 1/[σ^2^(*F*_o_^2^) + (0.0694*P*)^2^ + 1.9231*P*] where *P* = (*F*_o_^2^ + 2*F*_c_^2^)/3
*wR*(*F*^2^) = 0.106	(Δ/σ)_max_ < 0.001
*S* = 0.86	Δρ_max_ = 0.51 e Å^−^^3^
2580 reflections	Δρ_min_ = −0.59 e Å^−^^3^
172 parameters	Extinction correction: none
Primary atom site location: structure-invariant direct methods	Absolute structure: Flack (1983), with 1072 Friedel pairs
Secondary atom site location: difference Fourier map	Flack parameter: 0.340 (15)

### Special details

**Table:**

Geometry. All e.s.d.'s (except the e.s.d. in the dihedral angle between two l.s. planes) are estimated using the full covariance matrix. The cell e.s.d.'s are taken into account individually in the estimation of e.s.d.'s in distances, angles and torsion angles; correlations between e.s.d.'s in cell parameters are only used when they are defined by crystal symmetry. An approximate (isotropic) treatment of cell e.s.d.'s is used for estimating e.s.d.'s involving l.s. planes.
Refinement. Refinement of *F*^2^ against ALL reflections. The weighted *R*-factor *wR* and goodness of fit *S* are based on *F*^2^, conventional *R*-factors *R* are based on *F*, with *F* set to zero for negative *F*^2^. The threshold expression of *F*^2^ > σ(*F*^2^) is used only for calculating *R*-factors(gt) *etc*. and is not relevant to the choice of reflections for refinement. *R*-factors based on *F*^2^ are statistically about twice as large as those based on *F*, and *R*- factors based on ALL data will be even larger.

### Fractional atomic coordinates and isotropic or equivalent isotropic displacement parameters (Å^2^)

**Table:**

	*x*	*y*	*z*	*U*_iso_*/*U*_eq_	
Br1	0.49661 (7)	0.52137 (3)	0.02595 (3)	0.05940 (19)	
O1	0.3914 (4)	0.9390 (2)	0.17914 (19)	0.0495 (7)	
C8	0.1674 (5)	0.7808 (3)	0.2493 (2)	0.0306 (8)	
C17	0.2606 (5)	0.8175 (3)	0.3350 (2)	0.0334 (8)	
H17A	0.3496	0.7662	0.3548	0.040*	
H17B	0.3269	0.8812	0.3228	0.040*	
C2	0.4172 (5)	0.8448 (3)	0.1434 (2)	0.0363 (9)	
C11	−0.2109 (5)	0.7762 (4)	0.3180 (3)	0.0512 (11)	
H11A	−0.2768	0.7516	0.2650	0.061*	
H11B	−0.2999	0.7871	0.3662	0.061*	
C12	−0.0155 (7)	0.9143 (3)	0.3787 (3)	0.0495 (10)	
H12A	0.0472	0.9785	0.3646	0.059*	
H12B	−0.1034	0.9275	0.4270	0.059*	
C9	0.0205 (6)	0.8587 (3)	0.2208 (2)	0.0403 (9)	
H9A	0.0790	0.9237	0.2052	0.048*	
H9B	−0.0441	0.8333	0.1677	0.048*	
C7	0.3094 (5)	0.7656 (3)	0.1763 (2)	0.0307 (8)	
C14	0.0274 (6)	0.7365 (3)	0.4301 (2)	0.0439 (10)	
H14A	−0.0610	0.7469	0.4786	0.053*	
H14B	0.1175	0.6858	0.4499	0.053*	
C16	0.0668 (5)	0.6818 (3)	0.2726 (3)	0.0374 (9)	
H16A	0.0037	0.6562	0.2191	0.045*	
H16B	0.1559	0.6300	0.2913	0.045*	
C6	0.3380 (5)	0.6697 (3)	0.1394 (2)	0.0346 (8)	
H6	0.2673	0.6141	0.1594	0.042*	
C4	0.5731 (6)	0.7319 (4)	0.0433 (3)	0.0469 (10)	
H4	0.6633	0.7200	−0.0005	0.056*	
C1	0.4924 (7)	1.0210 (3)	0.1470 (3)	0.0544 (10)	
H1A	0.4601	1.0823	0.1800	0.082*	
H1B	0.4660	1.0309	0.0839	0.082*	
H1C	0.6219	1.0070	0.1547	0.082*	
C3	0.5459 (6)	0.8275 (3)	0.0770 (3)	0.0472 (11)	
H3	0.6155	0.8825	0.0549	0.057*	
C15	−0.0708 (5)	0.6981 (3)	0.3467 (3)	0.0428 (9)	
H15	−0.1328	0.6326	0.3604	0.051*	
C13	0.1216 (5)	0.8356 (3)	0.4087 (2)	0.0380 (9)	
H13	0.1854	0.8607	0.4629	0.046*	
C10	−0.1150 (6)	0.8754 (4)	0.2966 (3)	0.0472 (11)	
H10	−0.2068	0.9267	0.2777	0.057*	
C5	0.4671 (5)	0.6541 (3)	0.0743 (2)	0.0400 (9)	

### Atomic displacement parameters (Å^2^)

**Table:**

	*U*^11^	*U*^22^	*U*^33^	*U*^12^	*U*^13^	*U*^23^
Br1	0.0757 (3)	0.0442 (3)	0.0583 (3)	0.0164 (3)	0.0146 (3)	−0.00538 (19)
O1	0.0647 (19)	0.0356 (16)	0.0483 (16)	−0.0131 (14)	0.0155 (15)	−0.0039 (14)
C8	0.0306 (19)	0.030 (2)	0.0313 (19)	0.0015 (15)	0.0001 (14)	0.0022 (15)
C17	0.0330 (18)	0.038 (2)	0.0294 (18)	−0.0019 (16)	−0.0002 (15)	0.0021 (16)
C2	0.041 (2)	0.037 (2)	0.0314 (18)	−0.0044 (18)	0.0016 (16)	0.0021 (16)
C11	0.029 (2)	0.071 (3)	0.053 (3)	0.001 (2)	0.0018 (19)	0.000 (2)
C12	0.056 (2)	0.050 (3)	0.043 (2)	0.014 (2)	0.009 (2)	−0.0064 (17)
C9	0.040 (2)	0.045 (2)	0.0352 (17)	0.006 (2)	−0.0033 (17)	0.0085 (15)
C7	0.0307 (17)	0.034 (2)	0.0274 (17)	−0.0012 (15)	−0.0032 (14)	0.0003 (15)
C14	0.040 (2)	0.057 (3)	0.0337 (18)	0.005 (2)	0.0062 (17)	0.0110 (17)
C16	0.036 (2)	0.034 (2)	0.043 (2)	−0.0045 (16)	0.0017 (15)	0.0047 (18)
C6	0.035 (2)	0.037 (2)	0.0327 (18)	−0.0003 (16)	0.0008 (14)	0.0045 (16)
C4	0.045 (2)	0.057 (3)	0.039 (2)	−0.002 (2)	0.0116 (17)	−0.0006 (19)
C1	0.056 (2)	0.040 (2)	0.068 (3)	−0.010 (3)	0.000 (2)	0.0100 (19)
C3	0.050 (3)	0.051 (3)	0.040 (2)	−0.013 (2)	0.0087 (18)	0.0027 (19)
C15	0.037 (2)	0.046 (2)	0.046 (2)	−0.0059 (17)	0.0053 (17)	0.0051 (19)
C13	0.040 (2)	0.046 (2)	0.0274 (18)	0.0017 (17)	0.0017 (16)	−0.0017 (17)
C10	0.036 (2)	0.060 (3)	0.046 (2)	0.018 (2)	−0.0030 (18)	0.007 (2)
C5	0.045 (2)	0.040 (2)	0.0353 (18)	0.0066 (18)	0.0013 (17)	−0.0023 (16)

### Geometric parameters (Å, °)

**Table:**

Br1—C5	1.911 (4)	C9—H9B	0.9900
O1—C2	1.369 (5)	C7—C6	1.399 (5)
O1—C1	1.400 (5)	C14—C13	1.516 (6)
C8—C7	1.533 (5)	C14—C15	1.536 (6)
C8—C17	1.541 (5)	C14—H14A	0.9900
C8—C16	1.544 (5)	C14—H14B	0.9900
C8—C9	1.555 (5)	C16—C15	1.525 (6)
C17—C13	1.531 (5)	C16—H16A	0.9900
C17—H17A	0.9900	C16—H16B	0.9900
C17—H17B	0.9900	C6—C5	1.383 (5)
C2—C3	1.398 (5)	C6—H6	0.9500
C2—C7	1.405 (5)	C4—C5	1.373 (6)
C11—C10	1.523 (7)	C4—C3	1.375 (6)
C11—C15	1.524 (6)	C4—H4	0.9500
C11—H11A	0.9900	C1—H1A	0.9800
C11—H11B	0.9900	C1—H1B	0.9800
C12—C13	1.519 (6)	C1—H1C	0.9800
C12—C10	1.528 (6)	C3—H3	0.9500
C12—H12A	0.9900	C15—H15	1.0000
C12—H12B	0.9900	C13—H13	1.0000
C9—C10	1.535 (6)	C10—H10	1.0000
C9—H9A	0.9900		
			
C2—O1—C1	119.5 (3)	C15—C16—C8	111.5 (3)
C7—C8—C17	109.7 (3)	C15—C16—H16A	109.3
C7—C8—C16	112.4 (3)	C8—C16—H16A	109.3
C17—C8—C16	106.9 (3)	C15—C16—H16B	109.3
C7—C8—C9	111.4 (3)	C8—C16—H16B	109.3
C17—C8—C9	109.6 (3)	H16A—C16—H16B	108.0
C16—C8—C9	106.7 (3)	C5—C6—C7	121.4 (4)
C13—C17—C8	110.9 (3)	C5—C6—H6	119.3
C13—C17—H17A	109.4	C7—C6—H6	119.3
C8—C17—H17A	109.4	C5—C4—C3	118.5 (4)
C13—C17—H17B	109.4	C5—C4—H4	120.7
C8—C17—H17B	109.4	C3—C4—H4	120.7
H17A—C17—H17B	108.0	O1—C1—H1A	109.5
O1—C2—C3	121.6 (4)	O1—C1—H1B	109.5
O1—C2—C7	117.4 (3)	H1A—C1—H1B	109.5
C3—C2—C7	121.0 (4)	O1—C1—H1C	109.5
C10—C11—C15	109.1 (3)	H1A—C1—H1C	109.5
C10—C11—H11A	109.9	H1B—C1—H1C	109.5
C15—C11—H11A	109.9	C4—C3—C2	120.9 (4)
C10—C11—H11B	109.9	C4—C3—H3	119.6
C15—C11—H11B	109.9	C2—C3—H3	119.6
H11A—C11—H11B	108.3	C11—C15—C16	109.9 (3)
C13—C12—C10	109.3 (3)	C11—C15—C14	109.2 (4)
C13—C12—H12A	109.8	C16—C15—C14	109.4 (3)
C10—C12—H12A	109.8	C11—C15—H15	109.5
C13—C12—H12B	109.8	C16—C15—H15	109.5
C10—C12—H12B	109.8	C14—C15—H15	109.5
H12A—C12—H12B	108.3	C14—C13—C12	110.3 (3)
C10—C9—C8	110.1 (3)	C14—C13—C17	109.1 (3)
C10—C9—H9A	109.6	C12—C13—C17	109.7 (3)
C8—C9—H9A	109.6	C14—C13—H13	109.2
C10—C9—H9B	109.6	C12—C13—H13	109.2
C8—C9—H9B	109.6	C17—C13—H13	109.2
H9A—C9—H9B	108.2	C11—C10—C12	109.9 (4)
C6—C7—C2	116.7 (3)	C11—C10—C9	109.7 (4)
C6—C7—C8	120.5 (3)	C12—C10—C9	109.7 (3)
C2—C7—C8	122.8 (3)	C11—C10—H10	109.1
C13—C14—C15	109.1 (3)	C12—C10—H10	109.1
C13—C14—H14A	109.9	C9—C10—H10	109.1
C15—C14—H14A	109.9	C4—C5—C6	121.5 (4)
C13—C14—H14B	109.9	C4—C5—Br1	119.5 (3)
C15—C14—H14B	109.9	C6—C5—Br1	119.0 (3)
H14A—C14—H14B	108.3		

### Hydrogen-bond geometry (Å, °)

**Table:**

*D*—H···*A*	*D*—H	H···*A*	*D*···*A*	*D*—H···*A*
C9—H9A···O1	0.99	2.35	3.003 (5)	123
C17—H17B···O1	0.99	2.35	3.004 (4)	123
C4—H4···Cg^i^	0.95	2.66	3.568 (5)	161

Symmetry codes: (i) *x*−1/2, −*y*+1/2, −*z*+1.
